# Efficacy and Safety of Radiotherapy in Head and Neck Paragangliomas: A Retrospective 23-Year Analysis

**DOI:** 10.3390/jcm15031062

**Published:** 2026-01-29

**Authors:** Kimia Cepni, Meltem Dagdelen, Huseyin Oner, Bahar Cepni, Huriye Senay Kiziltan, Omer Erol Uzel

**Affiliations:** 1 Department of Radiation Oncology, Basaksehir Cam and Sakura City Hospital, University of Health Sciences, 34840 Istanbul, Turkey; 2 Department of Radiation Oncology, Cerrahpasa Medical Faculty Hospital, 34098 Istanbul, Turkey; meltem.dagdelen@iuc.edu.tr (M.D.); omeruzel@hotmail.com (O.E.U.); 3 Department of Radiation Oncology, Heidelberg University Hospital, 69120 Heidelberg, Germany

**Keywords:** paraganglioma, radiotherapy, IMRT, HNPG

## Abstract

**Background/Objectives**: Head and neck paragangliomas (HNPGs) are rare, typically benign, hypervascular tumors arising from neural crest cells. Although surgery is known as the primary treatment, radiotherapy (RT) is preferred for large and inoperable tumors. This study evaluated the results and safety of RT. **Methods**: Fifteen patients with radiologically or histologically confirmed HNPG treated with RT between 2001 and 2024 were retrospectively analyzed. Most patients received intensity-modulated radiotherapy (IMRT) with a median dose of 45 Gy. Treatment-related toxicities were graded according to CTCAE criteria. Local control (LC) was estimated with using Wilson score method. **Results**: With a median follow-up of 96 months, all patients achieved stable or improved symptomes, without local recurrence, resulting in a 100% LC rate. RT was well tolerated, with no acute or late toxicities ≥ grade 2. **Conclusions**: RT, particularly IMRT, provides excellent long-term LC with minimal toxicity in patients with HNPG. RT represents an effective and well-tolerated treatment option, especially for patients with unresectable disease or high surgical risk.

## 1. Introduction

Paragangliomas (PGs) are typically characterized as hypervascular neoplasms of low-grade malignancy, originating from neural crest-derived chief cells, which are located within the autonomic nervous system. These tumors can occur in various locations, including the head and neck, where they are often referred to as glomus tumors and can lead to significant clinical complications [1]. Approximately 3% of PGs occur in the head and neck area [2]. In the anatomical domain of the head and neck, the identified PGs occur approximately in 65% of cases within the carotid body, commonly referred to as carotid body neoplasms (CBT). Vagal paragangliomas arise from the autonomic component of the vagal nerve (cranial nerve X) [2]. HNPG primarily manifests in females, exhibiting a female to male ratio of 4:1 during the fifth and sixth decades of life [1].

The clinical course of glomus tumors frequently embodies the subtle and gradual manifestation of their associated symptoms. The prevalent manifestations include conductive hearing impairment and pulsatile tinnitus. Additional auditory indicators consist of otorrhea, hemorrhagic discharge, vertigo and the identification of a middle ear mass. Notably, pronounced otalgia is infrequently observed. The presentation of jugular foramen syndrome (paresis of the cranial nerves IX and X) is regarded as a pathognomonic feature for this neoplasm; however, it typically arises approximately one year after the initial symptoms, including auditory dysfunction and pulsatile tinnitus. The development of headaches, hydrocephalus, increased intracranial pressure, ataxia, and brainstem-associated symptoms may also arise [3].

PGs are predominantly benign, indolently proliferating neoplasms and were initially characterized by Von Haller in 1743 [4].

HNPGs arising in the petrous bone originate from the tympanic glomus or the jugular glomus. When these tumors cannot be differentiated, they are referred to as jugulo tympanic paragangliomas. Given the extensive spreading of the autonomic nervous structure, HNPG can arise at other locations, such as the ciliary glomus or laryngic glomus [5].

The HNPG compasses two known variants, particularly the sporadic variant and the familial variant. A notable thirty-five percent of all cases of HNPG are attributed to the familial variant [6]. The hereditary variant of HNPG exhibits a more pronounced correlation with genetic mutations. While genetic mutations may also arise de novo, the majority of patients presenting with genetic mutations typically possess a confirmed familial history [7].

Recently, a genetic alteration within the succinate dehydrogenase (SDH) gene complex has been correlated with Inherited Familial Paraganglioma Syndrome and is hypothesized to be present in a minimum of 30% of instances [8]. Owing to the significant correlation between genetic anomalies in HNPG and a favorable familial history, the manifestation of multiple neoplasms, and the relatively early age of onset, the implementation of screening protocols and evaluation by an imaging modality is necessary to establish the diagnosis [7].

While typically classified as benign hypervascular neoplasms, these tumors have the potential to metastasize to adjacent lymphatic nodes and remote organs. Intra-abdominal tumors exhibit a slight male predominance, whereas head and neck tumors demonstrate a greater prevalence among females [1]. Malignant HNPGs are observed in 6% to 19% of cases among male patients [9].

Differentiating HNPG from other lesions requires a systematic approach. Key mimics include schwannomas, which are hypovascular and lack early arterial-phase enhancement [1,10]; meningiomas, often showing hyperostosis and a dural tail; metastatic lymph nodes, usually avascular and without cranial nerve involvement [2]; and middle ear lesions such as adenomas, hemangiomas, and cholesteatomas, which may cause conductive hearing loss. Vascular anomalies like internal carotid artery aneurysms can also mimic glomus tumors, highlighting the importance of careful imaging to avoid misdiagnosis and potential hemorrhagic complications [4,10].

The accurate differentiation of HNPG from these entities relies heavily on advanced imaging modalities. Imaging procedures frequently used include B-mode sonography with color-coded Doppler sonography and computed tomography (CT) may prove beneficial for these individuals [10]. MRI, particularly when enhanced with contrast agents, continues to be regarded as the definitive method for evaluating tumor dimensions, vascular characteristics, and involvement of the skull base. Digital subtraction angiography (DSA) offers comprehensive vascular visualization and is notably advantageous for surgical strategizing or preoperative embolization procedures. Digital subtraction angiography and three-dimensional time-of-flight magnetic resonance angiography enable highly sensitive and specific assessment of vascular anatomy and flow characteristics in paragangliomas (PGs) [11,12].

Functional imaging techniques, such as PET/CT with ^68^Ga-DOTATATE or ^18^F-FDG, may aid in differentiating HNPGs from other disease entities and help detect multicentric or metastatic disease in genetic cases [8]. Historically, molecular imaging of this disease relied on I-123 MIBG combined with CT or MRI, while somatostatin receptor imaging was performed using In-111 DTPA pentetreotide SPECT/CT. Among DOTA-conjugated tracers, DOTATATE, DOTANOC, and DOTATOC are currently in clinical use, all exhibiting high affinity for somatostatin receptor subtype 2 [13].

The treatment of HNPG has evolved toward a patient-centered model, balancing the imperative of disease control with the preservation of functional capacity. The determination of therapeutic strategies is contingent upon various factors, including tumor localization, dimensions, multiplicity, genetic predisposition, and clinical manifestations.

Observation represents a valid methodology, particularly in geriatric patients or individuals presenting with asymptomatic, indolent neoplasms. Numerous contemporary studies indicate that a ‘monitor-and-evaluate’ strategy is appropriate for cases of indolent disease, thereby reducing unnecessary morbidity associated with surgical intervention or radiotherapy [14].

Sometimes chemotherapy using cyclophosphamide, vincristine, and dacarbazine (CVD protocol) is applied, particularly in progressive metastatic disease [15]. Targeted therapies, including tyrosine kinase inhibitors (e.g., sunitinib), are under investigation for SDHB-mutated cases and have shown promise in case series and early-phase trials [13]. Genetic testing is advised for all individuals, especially for those presenting with multifocal, early-onset, or hereditary neoplasms [8]. The identification of such mutations is crucial as it not only guides the therapeutic approach for the primary patient but also influences the screening protocols for their relatives [16,17].

Historically, surgery has been the primary treatment for HNPG; however, it is associated with substantial risks, including vascular injury leading to cerebrovascular events and significant cranial nerve deficits. These complications can result in permanent neurological, functional, and cosmetic morbidities such as hoarseness, aspiration, and the need for long-term airway or nutritional support. Preoperative embolization has been shown to reduce intraoperative blood loss, especially in highly vascular tumors [18]. Preoperative embolization remains controversial, with some studies suggesting reduced blood loss, while others do not recommend it for carotid body tumor (CBT) due to limited embolization of tumor-feeding vessels arising from the carotid adventitia. Surgically treated non-CBT patients had larger tumors and longer hospital stays, likely related to higher rates of cranial nerve deficits and postoperative complications [19,20].

Because the preferred way to handle resectable and symptomatic neoplasms is through surgical procedures. CBTs, for instance, are frequently categorized utilizing the Fisch or Shamblin classification system to anticipate the intricacies of the surgical procedure and associated vascular risks. The Fisch classification is commonly applied to temporal bone PGs, while the Glasscock–Jackson and Shamblin classifications are used for temporal PGs and carotid body tumors, respectively. According to the modified Fisch classification, early-stage tumors (Class A–B) are generally amenable to surgical resection with acceptable morbidity, whereas advanced lesions (Class C–D), particularly those with carotid canal or intracranial extension, are associated with significantly higher risks of cranial nerve injury and vascular complications. Therefore, in advanced-stage disease, radiotherapy or observation is often favored over primary surgery to achieve tumor control while minimizing treatment-related morbidity [6,10,21].

Nevertheless, surgical interventions in proximity to the skull base, particularly those addressing jugular or vagal paragangliomas, entail considerable risk to the cranial nerve structures. Advancements such as transoral robotic surgery (TORS) and the application of neuromonitoring techniques have been investigated to mitigate postoperative morbidity [22].

It is now recognized that up-front surgical management is often not the optimal approach for HNPG. Multiple factors should be taken into consideration when deciding the most appropriate treatment, such as age, comorbidities, prior treatment, likelihood of developing additional lesions, and preexisting cranial nerve deficiencies. Primary surgery should be considered for young, otherwise healthy patients with small- to medium-sized CBTs, particularly in cases of secreting tumors, suspicion of malignancy, rapid tumor growth, or worsening symptomatology, as well as when radiotherapy is not feasible. Conversely, in the remaining cases, active surveillance with serial imaging may be offered to avoid unnecessary morbidity while maintaining acceptable tumor control [22,23].

RT provides superior local control, particularly in neoplasms that are not amenable to surgical intervention or in individuals who possess unfavorable surgical profiles. While SRS or Stereotactic Body Radiotherapy (SBRT) are generally preferred for HNPG of 4 cm and smaller, hypofractionated or conventional fractionated RT methods are preferred for larger tumors to avoid increasing complications. Stereotactic radiosurgery (SRS) combined with intensity-modulated radiotherapy (IMRT) provides superior spatial conformity and lessens the occurrence of undesirable effects [3,24]. Recent retrospective studies report local control rates above 90% with modern techniques [24]. A large Bayesian meta-analysis comparing surgery and stereotactic radiosurgery (SRS) for jugular paragangliomas demonstrated excellent long-term tumor control with both modalities; however, SRS was associated with significantly lower rates of treatment-related morbidity. Although SRS was used for larger tumors, it was associated with lower recurrence rates and fewer complications particularly cerebrospinal fluid leakage, dysphagia, and lower cranial nerve palsies, highlighting its more favorable toxicity profile compared with surgery. These findings support the role of SRS as an effective and less morbid alternative to surgical management, especially in selected patients. SRS is generally preferred for tumors smaller than 3 cm, while conventionally fractionated external beam radiotherapy (EBRT) is recommended for larger tumors or those with extracranial extension [25,26].

In this study, treatment outcomes were evaluated for patients with paraganglioma who underwent radiotherapy for head and neck lesions.

## 2. Materials and Methods

A total of 15 patients diagnosed with paraganglioma who underwent RT between 2001 and 2024 were retrospectively evaluated. Fifteen patients, confirmed radiologically (n = 14) or histopathologically (n = 1), were included this study. Demographic and clinical characteristics are summarized in Table 1 and Table 2, and Supplemented Table S1.

**Figure 1:**
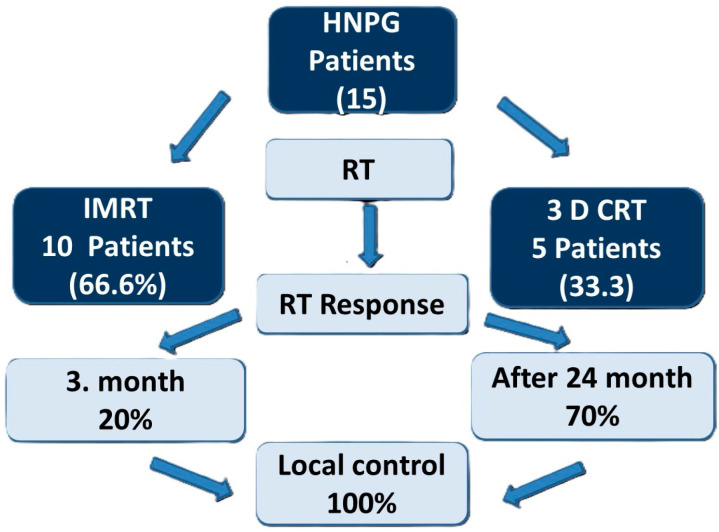
Flow chart of this study.

Diagnosis was established by MRI in 9 patients, MRI and CT in 4 patients, MR and angiography in 2 patients. In one patient with a history of surgery, histopathological verification was available. All patients underwent MRI for treatment planning. MRI served as the primary imaging modality to evaluate tumor extent, cranial nerve involvement and for RT planning. Flow chart of the study is shown in Figure 1.

All patients received external beam radiotherapy (EBRT). IMRT was used in 10 patients, while three-dimensional conformal radiotherapy (3D-CRT) was applied in 5 patients. The median total dose administered was 45 Gy (range: 25–54 Gy) (Table 2). Gross tumor volume (GTV) was defined as the visible tumor mass identified through imaging. In patients treated with image-guided radiotherapy (IGRT), a margin of 3 mm was added to the GTV to define the planning target volume (PTV).

Acute and late treatment-related toxicities were retrieved from patient records with auditory and neurologic tests (before and 1 year after RT) graded according to the Common Terminology Criteria for Adverse Events (CTCAE). Tinnitus and hearing loss were considered radiation-related adverse effects if they were not present at the onset of the disease, as none of the patients showed disease progression.

The estimated LC rate was determined using the Wilson method, with a 95% CI due to the limited sample size.

## 3. Results

A total of 15 patients diagnosed with HNPG and treated with RT were retrospectively evaluated. The mean age at diagnosis was 53 years (range: 27–75). The majority of the patient group consisted of females (13 females, 87%; 2 males, 13%).

The most frequent indication for RT was unresectable disease. The median follow-up time was 96 months (range: 23–124), only one patient who developed recurrence after surgery was also included in this study. Since no local recurrences were observed during the follow-up period, the LC rate after RT was 100%.

Only one patient had a history of surgical resection. The patient had undergone surgery twice in the past and was referred for RT due to recurrence. The other patient was diagnosed with bilateral PG, underwent surgery on the left side, and received primary RT for the right side.

RT was delivered using IMRT in ten patients (66.6%) and 3D-CRT in five patients (33.3%). The median prescribed radiation dose was 45 Gy (interquartile range not available; range, 25–54 Gy), administered in a median of 25 fractions (range, 5–28). The median target tumor volume was 51 cm^3^ (range, 14–163 cm^3^).

The median PTV was 55 cm^3^ (range, 22–174 cm^3^) (Table 3).

A volumetric analysis demonstrated a median tumor reduction at 3 months of 20% (range, 0–50%), and 70% at >24 months (range, 50–100%). In addition to the unidimensional assessment based on RECIST version 1.1, volumetric tumor analysis was performed to more accurately characterize treatment-related changes. Tumor volumes were calculated on pre- and post-radiotherapy MRI through manual contouring of the lesion on consecutive axial slices. Total tumor volume was derived by summing the contoured areas and multiplying by the slice thickness. Percentage volumetric change was determined by comparing baseline and follow-up tumor volumes. Volumetric analysis was used as a complementary approach to RECIST, particularly given the typically stable radiological appearance and irregular morphology of head and neck paragangliomas. (Table 4).

LC was achieved in all patients, corresponding to a 100% LC rate. Using the Wilson method, the estimated LC rate was 100% with a 95% CI of 79.6–100%, reflecting the limited sample size despite the absence of local failure events (Table 4).

Clinical outcomes were heterogeneous despite excellent local control. Stable, improved and resolved symptoms were in five patients (33.3%) respectively. Among the ten patients with persistent symptoms, four had been treated with 3D-CRT. Given that only five patients in total received 3D-CRT, persistent symptoms were observed in 80% of this subgroup. In the IMRT cohort, persistent symptoms were reported in six of ten patients (60%).

Overall, RT resulted in durable local tumor control with progressive radiologic response over time, as reflected by increasing median response percentages, while clinical benefit in terms of symptom relief was achieved in a substantial subset of patients (Table 4).

The detected toxicities related to RT were mostly mild (grade 1–2) and limited to symptoms such as mucositis, dysphagia, and alopecia in three patients (20%). Tinnitus did not relief after RT in four patients (26.6%). Similarly, hearing loss did not improve in one patient (6.6%). Tinnitus and hearing loss were considered radiation-related adverse effects if they were not present at the onset of the disease, as none of the patients showed disease progression.

RT was well tolerated in all patients, and no ≥grade 2 acute or late toxicity was reported. The most common presenting symptoms were tinnitus, a sense of fullness in the ear, and headache.

## 4. Discussion

The present study retrospectively analyzed the clinical outcomes of 15 patients diagnosed with HNPG who received RT, revealing exceptional LC accompanied by minimal adverse effects. In alignment with the prevailing literature, our research underscores the significance of RT as a feasible and efficacious therapeutic modality, especially for neoplasms that are not suitable for surgical resection or in scenarios where surgical intervention entails considerable morbidity risks.

In our cohort, one patient had previously undergone embolization; however, the lesion continued to receive vascular supply, necessitating referral for RT. Another patient underwent two surgical interventions, both followed by recurrence. At the time of the latest recurrence, the patient presented with facial nerve paralysis and was referred for RT. In one patient with bilateral paragangliomas, surgical intervention was performed on one side, while the contralateral lesion was effectively managed with RT.

RT provides superior LC, particularly in neoplasms that are not suitable for surgical intervention or in patients with unfavorable surgical profiles. Recent retrospective studies report LC rates above 90% with modern RT techniques [24,27].

An alternative approach for large lesions that cannot be completely resected is surgical debulking followed by SRT. However, in a relatively large retrospective cohort, tumor control achieved with SRT alone was reported to be superior to that obtained with combined-modality treatment. Nevertheless, the addition of SRT to residual disease may still reduce surgery-related morbidity compared with surgery alone [27,28]. Long-term outcomes of paraganglioma treatment using stereotactic radiosurgery and intensity-modulated radiotherapy have been investigated in some studies in upfront, adjuvant, and salvage settings [29,30].

RT, including IMRT and 3D-CRT, provided favorable outcomes in this cohort, achieving stable disease and symptom management. IMRT’s precise targeting capabilities allow for the sparing of surrounding critical structures, thereby reducing side effects and improving patient quality of life, as observed in our patient cohort and supported by several recent studies [31]. A retrospective study by Gilbo et al. reported a LC rate exceeding 90% using IMRT for head and neck paragangliomas, further validating our findings [32]. SRS provides superior spatial conformity and lessens the occurrence of undesirable effects [3,24].

The median radiation dose in our study was 45 Gy, aligning with recommended doses ranging from 45 to 54 Gy reported in the literature, which balance tumor control and toxicity profiles effectively [24].

In a study comparing the results of radiotherapy and surgery, Mendenhall et al. applied 30–70 Gy RT to inoperative PG patients. Although the patients in the RT group had large tumors, similar results were reported compared to surgery (73% vs. 78%) [33]. Kim et al., in a study investigating the optimal RT dose, reported relapse rates of 1% for doses above 40 Gy and 25% for doses below 40 Gy. Therefore, the optimal RT dose was accepted as 45 Gy [34]. Our results demonstrate that doses within this range offer excellent disease control with acceptable side effects.

PG tumors may appear unresponsive on the first MR images following radiotherapy; however, it is known that these tumors shrink over a period of up to 2 years. RT-induced fibrosis may appear as a mass in the tumor. Therefore, a stable mass can be interpreted as a successful outcome. The response rates in our clinical study are similar to those in the literatüre. Volumetric analysis was used as a complementary method to RECIST, particularly given the typically stable radiological appearance and irregular morphology of HNPG [35]. Therefore, volumetric evaluation was performed in the present study to more clearly demonstrate treatment-related tumor shrinkage. Based on volumetric analysis, a median tumor volume reduction of 20% was observed at 3 months, increasing to 70% after 24 months. In contrast, several previous studies have assessed response primarily using changes in tumor diameter. The comparatively longer median follow-up period in our cohort, together with the use of volumetric assessment, may explain why response rates beyond 24 months appeared more favorable than those reported in the literature [33,36,37].

In our patient cohort, clinical symptoms either stable, improved or resolved in 33.3% of patients consistent with findings reported in the literature [36,38].

Long-term cure rates are comparable between surgery and RT. Kyrch et al. reported a tumor control rate of 92% associated with EBRT [33,36,37]. The results of this study showed that 100% LC was achieved, consistent with the literature.

The absence of significant acute or late toxicity (≥grade 2) in our patients underscores RT’s favorable safety profile, in alignment with data from larger patient cohorts [9]. The minimal toxicity is particularly beneficial considering surgical approaches near critical neurovascular structures that carry a high risk of complications, including cranial nerve deficits. Even though most HNPGs are typically considered benign, malignant changes or metastatic spread has been reported in up to 19% of the cases [9].

The median follow-up period in our study was approximately 96 months, aligning with or exceeding several previously published studies, and providing robust evidence of sustained LC [24]. Long-term control without significant toxicity observed in this cohort further supports the efficacy and safety of RT in managing HNPG, particularly when surgical interventions are contraindicated or present excessive risk.

In this study, conventionally fractionated RT were employed due to a median tumor volume of 51 cm^3^. Despite the relatively large tumor sizes, 100% local control (LC) was achieved.

Strengths and Limitations: One of the primary strengths of this study is the long follow-up period, with a median duration of 96 months, allowing for a robust evaluation of long-term LC. Additionally, toxicity was prospectively analyzed using standardized CTCAE criteria, enhancing the reliability of safety data. Lastly, the homogeneous patient population with head and neck tumors increases the internal validity when interpreting outcomes.

Several limitations must be acknowledged. The retrospective design limits control over confounding factors, and the small patient cohort. Moreover, the absence of patient-reported outcome measures restricts the evaluation of functional and quality-of-life endpoints. However, due to the rarity of HNPG tumors, the preference for surgery, and the referral of patients with large tumors for radiotherapy, many studies have included only a small number of patients. Therefore, we believe that a cohort of 15 patients is sufficient for this study.

## 5. Conclusions

In conclusion, RT demonstrates excellent efficacy and tolerability in the management of head and neck paragangliomas comparable to surgery, even for larger tumors. The integration of genetic insights into treatment and surveillance strategies offers potential for personalized patient care or treatments like somatostatin analogs. Appropriate patient selection and careful consideration of tumor biology, receptor expression, and disease distribution are essential when choosing between these modalities, particularly in cases where both are feasible. Understanding their respective efficacy, safety, and toxicity profiles enables optimization of treatment outcomes while minimizing adverse effects. In addition to established therapies, emerging radiotherapeutic strategies offer promising perspectives that may further refine individualized treatment approaches and improve outcomes for patients with paraganglioma in the near future. Future prospective studies with larger patient cohorts and longer follow-up periods are warranted to validate these findings and optimize RT and other therapeutic strategies.

## Figures and Tables

**Table 1:** Clinical Characteristics of HNPG Patients.

Characteristic	Value
**Age (years), median (range)**	53.0 (37–72)
**Sex, n (%)**	
Female	12 (85.7)
Male	2 (14.3)
**Tumor location, n (%)**	
Right Jugular	5 (33.3)
Left Jugular	6 (40)
Jugular-CPA/CPA	2 (1.3)
Right Jugulotympanic	1 (0.6)
Bilateral Carotid	1 (0.6)
**Presenting symptoms, n (%)**	
Tinnitus/Bu ing	8 (53.3)
Hearing impairment/Loss	6 (40)
Di iness	3 (0.2)
Facial paralysis	1 (0.6)
Headache	1 (0.6)
Other	4 (26.6)

**Table 2:** RT parameters and clinical outcomes in HNPG patients.

Patient Number	RT Technique	Dose cGy	Fraction Size	PTV, cm^3^	Clinical Outcome	Presenting Symptomes	RT Response, 3 Month (%)	RT Response, >24 Month (%)	LC *
1	3D-CRT	5040	28	50	Stable	Tinnitus	20	70	100
2	3D-CRT	5040	28	30	Stable	Tinnitus	30	80	100
3	3D-CRT	5040	28	95	Stable	Tinnitus	10	70	100
4	IMRT	5040	28	60	Improved	Tinnitus	20	50	100
5	3D-CRT	4500	25	20	Resolved	-	20	80	100
6	IMRT	4500	25	85	Improved	Facial paralysis	10	50	100
7	IMRT	4500	25	170	Resolved	-	20	60	100
8	3D-CRT	4500	25	80	Stable	Throat and ear pain	10	50	100
9	IMRT	4500	25	25	Improved	Hearing loss	20	60	100
10	IMRT	4500	25	75	Improved	Tinnitus	20	70	100
11	IMRT	2500	5	25	Stable	Hearing loss, hoarseness	30	60	100
12	IMRT	5400	25	45	Resolved	-	30	100	100
13	IMRT	4500	25	50	Improved	Tinnitus	10	60	100
14	IMRT	5400	27	55	Resolved	-	20	80	100
15	IMRT	5000	25	65	Resolved	-	20	70	100

RT response: Determined as volumetric changes with RECIST Version 1.1 of HNPG tumors; LC: local control; PTV: Planning target volume; RT: Radiotherapy. *: Last follow-up, month.

**Table 3:** RT parameters of HNPG patients.

Variable	Median/n (%)
RT technique	IMRT: 10 (66.6%)
	3D-CRT: 5 (33.3%)
Dose, cGy	4500 (2500–5400)
Fractions	25 (5–28)
PTV, cm^3^	55 (20–172)
GTV, cm^3^	51 (14–163)

**Table 4:** Summary of clinical outcomes of HNPG patients.

Variables	Values n (%)
Local control	15 (%100)
RT response, 3 Month (%)	20 (0–50)
RT response, >24 Month (%)	70 (50–100)
Clinical Outcome (Symptoms)	Stable *: 5 (33.3%)
	Improved: 5 (33.3%)
	Resolved: 5 (33.3%)

* Persistent tinnitus, hearing loss, facial paralysis.

## Data Availability

The datasets analyzed during the current study are not publicly available due to institutional policy but are available from the corresponding author on reasonable request.

## Supplementary Materials

The following supporting information can be downloaded at: https://www.mdpi.com/article/10.3390/jcm15031062/s1, Table S1: Clinical characteristics of HNPG patients.

## Abbreviations

The following abbreviations are used in this manuscript:

HNPGs	Head and neck paragangliomas
RT	radiotherapy
IMRT	intensity-modulated radiotherapy
LC	Local control
PGs	paragangliomas
CBT	carotid body neoplasms
SDH	succinate dehydrogenase
CT	computed tomography
DSA	Digital subtraction angiography
TORS	transoral robotic surgery
SBRT	Stereotactic Body Radiotherapy
SRS	Stereotactic radiosurgery
EBRT	external beam radiotherapy
GTV	Gross tumor volume
IGRT	image-guided radiotherapy
PTV	planning target volume
CTCAE	Common Terminology Criteria for Adverse Events
