# Supplementary material for: Efficacy and Safety of Radiotherapy in Head and Neck Paragangliomas: A Retrospective 23-Year Analysis
Source: J Clin Med. 2026 Jan 29;15(3):1062. doi: 10.3390/jcm15031062 (PMC12897705; doi:10.3390/jcm15031062)
Supplement: Supplementary file 1 [file jcm-15-01062-s001.zip › jcm-4091554-supplementary.pdf]

Table S1. Clinical characteristics of HNPG patients.

Patient number	Age	Sex	Tumor Location	Presenting Symptoms
1	46	F	Left CPA–jugular foramen	Tinnitus
2	50	F	Right jugulo tympanic-internal auditory canal	Tinnitus
3	39	F	Left jugular bulb	Tinnitus
4	55	F	Left jugular foramen	Tinnitus, buzzing
5	51	F	Right jugular	Hearing impairment
6	65	F	Recurrent left CPA	Facial paralysis, tinnitus, hearing loss
7	53	F	Left jugulodigastric fossa	Dizziness, tinnitus, hearing loss
8	72	F	Left jugular	Throat and ear pain
9	70	F	Left jugular foramen	Tinnitus, hearing loss
10	53	F	Left jugular foramen	Hearing loss, dizziness
11	68	F	Right jugular foramen	Hearing loss, hoarseness
12	63	M	Right jugular foramen	Neck swelling
13	30	F	Bilateral carotid body	Bilateral tinnitus
14	37	F	Right jugular fossa	Dizziness
15	41	M	Right jugular fossa	Headache

F: Female M: Male CPA: Cerebello pontine angle
